# An evaluation of the impact of aggressive hypertension, diabetes and smoking cessation management on CVD outcomes at the population level: a dynamic simulation analysis

**DOI:** 10.1186/s12889-019-7429-2

**Published:** 2019-08-14

**Authors:** John Pastor Ansah, Ryan Leung Hoe Inn, Salman Ahmad

**Affiliations:** 1 Signature Program in Health Services and Systems Research, Duke-NUS Medical School, 8 College Road, Singapore, 169857 Singapore; 2 National University of Singapore, Residential College 4, 6 College Avenue East, University Town, Singapore, 138614 Singapore; 3 Department of Mechanical Engineering, National University of Singapore, 9 Engineering Drive 1, Singapore, 117575 Singapore

**Keywords:** CVD, Chronic disease management, System dynamics, Singapore

## Abstract

**Background:**

Evidence from randomized control trials suggest that coupled with smoking cessation interventions, CVD events can be reduced significantly if hypertension and diabetes patients are properly managed, raising practical what-if questions at the population level. This research aims to develop a dynamic simulation model using the systems modelling methodology of system dynamics, to evaluate the medium to long-term impact of hypertension and diabetes management, as well as smoking cessation intervention on CVD events, CVD deaths and post-CVD population.

**Methods:**

The systems modelling methodology of system dynamics was used to develop a simulation model to evaluate the impact of aggressive hypertension, diabetes and smoking cessation management on CVD outcomes at the population level.

**Result:**

The insights from this research suggest that despite that at the individual level, hypertension management is associated with the highest risk reduction for CVD (50%) compared to diabetes and smoking (20%) and is also the most prevalent risk factor, at the population level, diabetes management interventions are projected to have higher impact on reducing CVD events compared to hypertension management or smoking cessation interventions. However, a combined intervention of diabetes and hypertension management, as well as smoking cessation has the most impact on CVD outcomes.

**Conclusion:**

Due to aging population and the increasing prevalence of chronic conditions in Singapore, the number of CVD events in Singapore is projected to rise significantly in the near future—hence the need for proactive planning to implement needed interventions. Findings from this research suggest that CVD events and its associated deaths and disabilities could be reduced significantly if diabetes and hypertension patients are aggressively managed.

**Electronic supplementary material:**

The online version of this article (10.1186/s12889-019-7429-2) contains supplementary material, which is available to authorized users.

## Background

Cardiovascular disease (CVD) is one of the four leading causes of death around the world [1–4]. In 2012, around 17.5 million deaths were attributed to CVDs—of which an estimated 7.4 million were due to heart attacks (ischaemic heart disease) and 6.7 million were due to strokes [5]. CVDs, particularly stroke, are responsible not only for mortality but a significant number of long-term physical disability and hospitalization [6], thus lower quality of life and increasing medical costs.

Evidence from randomized control trials suggesting that CVD events can be reduced significantly if hypertension [7–9] and diabetes [10, 11] patients are properly managed and smoking cessation interventions are implemented, raises practical what-if questions at the population level, which can be examined through modelling and simulation. Thus, this research aims to develop a dynamic simulation model that allows for the evaluation of the medium to long-term impact of hypertension, diabetes and smoking cessation management interventions on CVD outcomes, specifically CVD events, CVD deaths and post-CVD population, at the population level in Singapore. Coupled with the prevalent issue of rapid population aging, Singapore also faces the concern of a growing number of people developing hypertension [12] and diabetes [13]. As of 2010, the prevalence of hypertension stood at 23.5% [14] compared to other developed countries; 33.4% in 2016 in USA [15]. 17.8% in 2017 for Canada [16], 48.9% in Japan [17], and 29.1% in 2016 for South Korea [18]. Likewise, the prevalence of diabetes in Singapore was 11.3% in 2010 [14] compared to 12.6% in USA as of 2016 [15], 7.0% in Canada in 2016 [19], 7.9% in Japan in 2010) [20] and 13.7% in South Korea as of 2010 [21]. The smoking rate is estimated to be 13% in Singapore compared to 20.9% in the USA [22], 13.7% in Canada [22], 20.0% in Japan [22] and 22.8% in South Korea [22]. If not adequately managed, such risk factors can become complicated and increase the risk of CVD events. Having an advanced understanding of the likely impact of diabetes, hypertension and smoking cessation management interventions on CVD outcomes through the translation of evidence into quantifiable numbers will in turn inform clinical interventions and health policy.

Modeling and simulation methods applied to CVDs include Markov Model, Agent-Based Modelling, and System Dynamics [23]. Markov models are a popular method of application [24] to project the future prevalence of CVD using risk factor trends derived from population-based surveys. In addition, Markov models have also been used to study the effects of targeted policy interventions on CVD, such as the study of dietary changes on future CVD events in the United Kingdom (UK) and the impact of Argentina’s national tobacco control law on CVD events [25]. Li Y, Kong and colleagues [26] have applied agent based modeling to study the impact of lifestyle interventions on the future prevalence of CVD in the United States [US], while Kruzikas DT and colleagues [27] used the same modeling approach to estimate the effect of health care system investment on CVD in India. System dynamics methodology has been used to evaluate impact of CVD interventions [28] The most prominent utilisation of system dynamics modelling on the epidemiology of CVD is the Prevention Impacts Simulation Model [PRISM] [29]. PRISM is a deterministic compartmental system dynamics model developed to study the effects of risk factors of CVD using population-level data and CVD risk engine [30]. Interest in the application of SD for CVD epidemiology studies has been on the rise as PRISM was adopted in New Zealand [31], and expanded to examine the impact of various CVD intervention policies [32–36].

## Methods

Based on publicly available data sets from the Ministry of Health, the systems modelling methodology of system dynamics [37] was used to create the simulation models for evaluating the impact of an aggressive hypertension and diabetes management and smoking cessation interventions on CVD events, CVD deaths and post-CVD population in Singapore. The system dynamics methodology consists of interacting sets of differential and algebraic equations developed from a broad range of relevant empirical data [23]. System dynamics models help policymakers to improve their ability to anticipate the likely impact of interventions over time on dynamically complex conditions *in-silico*, where pathways from interventions to outcomes may be indirect, delayed and possibly affected by nonlinearities or feedback loops [38]. The system dynamics methodology has been used effectively to address dynamically complex issues in healthcare [39–41], health policy [42] and social policy [43–45] including CVD [31, 35, 36, 46].

### The model structure

The model consists of two linked sub-models: the risk factor sub-model and CVD sub-model. The risk factor sub-model comprises of three sub-models—diabetes, hypertension, and smoking, while the CVD sub-model applies the *Framingham Risk Engine* to project CVD events and disabilities associated with CVD. Because the model structures for diabetes and hypertension are similar, a single model structure with subscripts/arrays is presented. The model presented herein was developed as follows: first, a conceptual model was developed that simulated the behaviour of key outcomes using available data and information from literature. Next, the conceptual model was presented to clinicians with expert knowledge on CVD to verify the model structure and its assumptions regarding causal relationships. Following verification, the model was simulated, base-case scenarios was developed with other alternative policies. Therefore, the model is grounded on current knowledge and available evidence on the risk factors of CVD and interventions to prevent CVD events.

#### Risk factor model

**Fig. 1:**
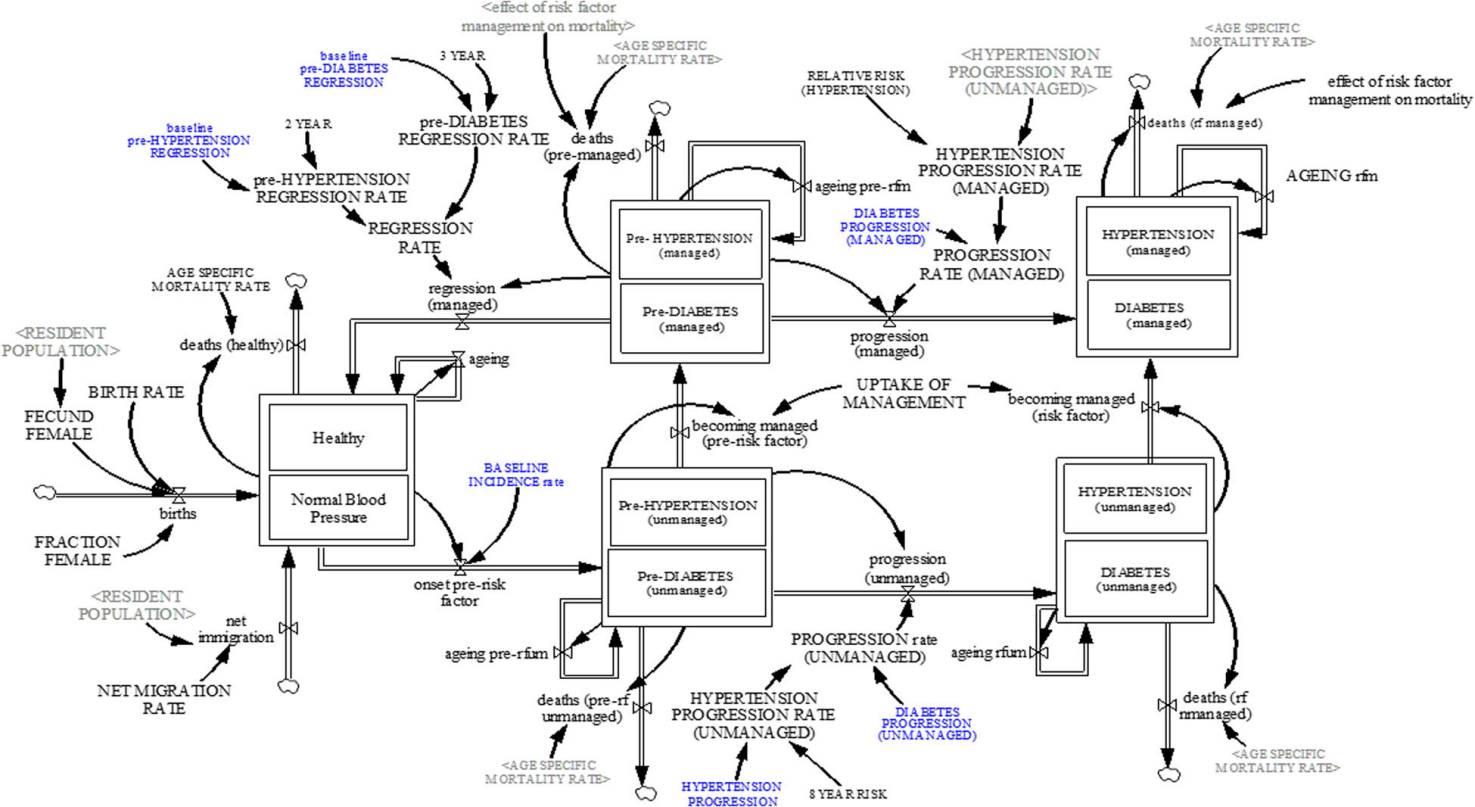
Risk factors sub-model (diabetes, and hypertension)

**Fig. 2:**
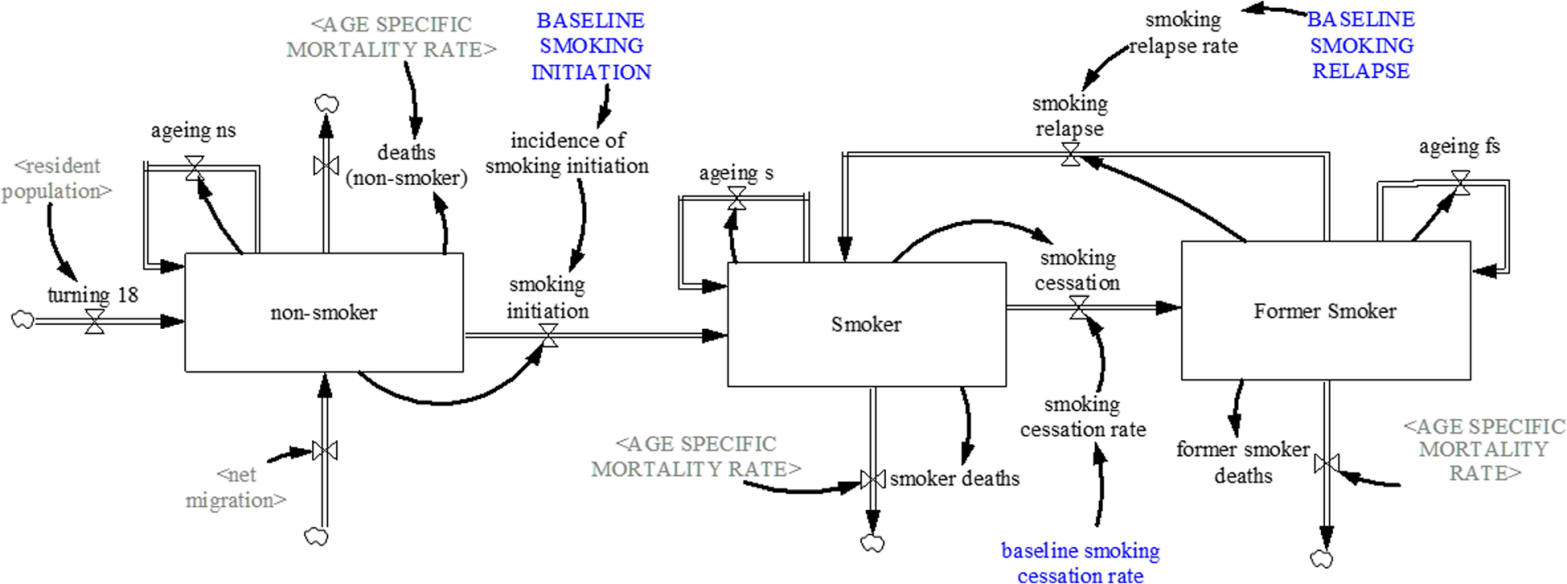
Smoking sub-model

Figure 1 shows the model structure of diabetes, and hypertension; whereas the smoking sub-model is shown in Fig. 2. Each sub-model is briefly explained:

#### Diabetes sub-model

The diabetes sub-model (see Fig. 1) projects the prevalence of diabetes within the population of Singapore. To project the prevalence of diabetes, the Singapore population was disaggregated into three health states—*healthy*, *pre-diabetes*, *diabetes.* These health states were further disaggregated by age (single age cohorts from age 18 to age 100 and older) and gender (male, female)*.* For the purpose of this model, the healthy state, refers to individuals with normal glycaemic levels of Haemoglobin A1C (HbA1C) below 5.7%. The pre-diabetes state is defined as individuals with HbA1C between 5.7–6.4%; whereas HbA1C > 6.4% is considered as the diabetes state. The pre-diabetes and diabetes health states are further divided into two groups—managed and unmanaged. The managed population is defined as individuals with pre-diabetes or diabetes receiving adequate behavioural or pharmacological care and treatment to manage the condition; whereas unmanaged pre-diabetes and diabetes are individuals not receiving treatment.

The healthy population increases through births, net migration and pre-diabetic individuals becoming healthy, and decreases as individuals transition from healthy state to pre-diabetes state and deaths. Births is determined by fecund female population (age 15—age 49) and fertility rate [47, 48]; while net migration is estimated through calibration. Similarly, deaths among the healthy, pre-diabetes and diabetes population is determined by age-specific mortality rate from life tables [47], adjusted by the effect of management/treatment on mortality for the managed diabetes and prediabetes population. The transition from healthy to pre-diabetes state is determined by a transition rate from literature [49], and the transition from pre-diabetes state to healthy state is determined by a transition rate from available evidence [50]. The pre-diabetic population increases via incidence of pre-diabetes and decreases via deaths and transition from pre-diabetes to diabetes, as well as with the transition from pre-diabetes to healthy state. The transition from pre-diabetes to diabetes is determined by a transition rate derived from literature [49]. Lastly, the diabetes population increases through the incidence of diabetes and decreases through deaths. For the pre-diabetes and diabetes population, becoming managed is dependent on identification via screening and uptake of treatment.

#### Hypertension sub-model

The hypertension sub-model (see Fig. 1) projects the prevalence of hypertension in Singapore by age (18 years and older) and gender. Akin to the diabetes sub-model, the population of Singapore was divided into three mutually exclusive health states: normal blood pressure, prehypertension, and hypertension. For the purpose of this model, hypertension state is defined as individuals with systolic blood pressure > 140 mm of mercury (mmHg) or diastolic blood pressure > 90 mmHg. Prehypertension state is defined as individuals with systolic blood pressure between 120 and 139 mmHg or diastolic blood pressure between 80 and 89 mmHg. Lastly, normal blood pressure is defined as individuals with a systolic blood pressure < 120 mmHg or diastolic blood pressure < 80 mmHg. To assess the impact of hypertension management on CVD events, the population in the prehypertension and hypertension states were further divided into managed and unmanaged. The prehypertension and hypertension managed state refers to individuals receiving prehypertension or hypertension behavioural or pharmacological treatment, whereas the unmanaged prehypertension and hypertension state consists of individuals receiving no treatment.

Within the hypertension sub-model, the normal blood pressure population increases via births, net migration and transition from prehypertension to normal blood pressure state, and decreases via deaths and transition from normal blood pressure to prehypertension. Births are determined by the female population between ages 15 to 49 and fertility rate [47, 48]; while deaths are determined by age-specific mortality rate from life tables [47]. The transition from normal blood pressure to prehypertension [incidence of prehypertension], prehypertension to normal blood pressure [regression of prehypertension] and prehypertension to hypertension [incidence of hypertension] are determined by transition rates derived from current literature [51–53]. The prehypertension population increases by incidence of prehypertension and decreases by deaths and transition from prehypertension to hypertension and prehypertension to normal blood pressure. Deaths among the prehypertension and hypertension population are determined by age-specific mortality rates [47], adjusted by the effect of management/treatment on mortality for the managed hypertension and prehypertension population. Lastly, the hypertension population increases via incidence of hypertension and decreases via deaths.

#### Smoking sub-model

The smoking sub-model (see Fig. 2) projects the prevalence of smoking among the Singaporeans 18 years and older. The smoking sub-model disaggregates the population into three smoking states—non-smokers, current smokers, and former smokers. Non-smokers are defined as individuals who have never smoked, while current smokers are individuals who have smoked regardless of frequency. Former smokers are individuals who have a history of smoking, but have not done so for six months prior to the interview [54].

The non-smokers population can increase through net migration or as individuals become 18 years old and transition into the non-smokers population, and decreases via smoking initiation and deaths. Since the population of interest is 18 and above, we assume that those turning18 first enter the non-smokers group before they become smokers, and that there are no current or former-smokers among those turning 18. The smoker’s population increases via smoking initiation and relapse of former smokers, and decreases through smoking cessation and deaths. Smoking initiation rate (model calibration), cessation rate [55] and relapse rate [56] were derived from literature as cited.

#### CVD sub-model

**Fig. 3:**
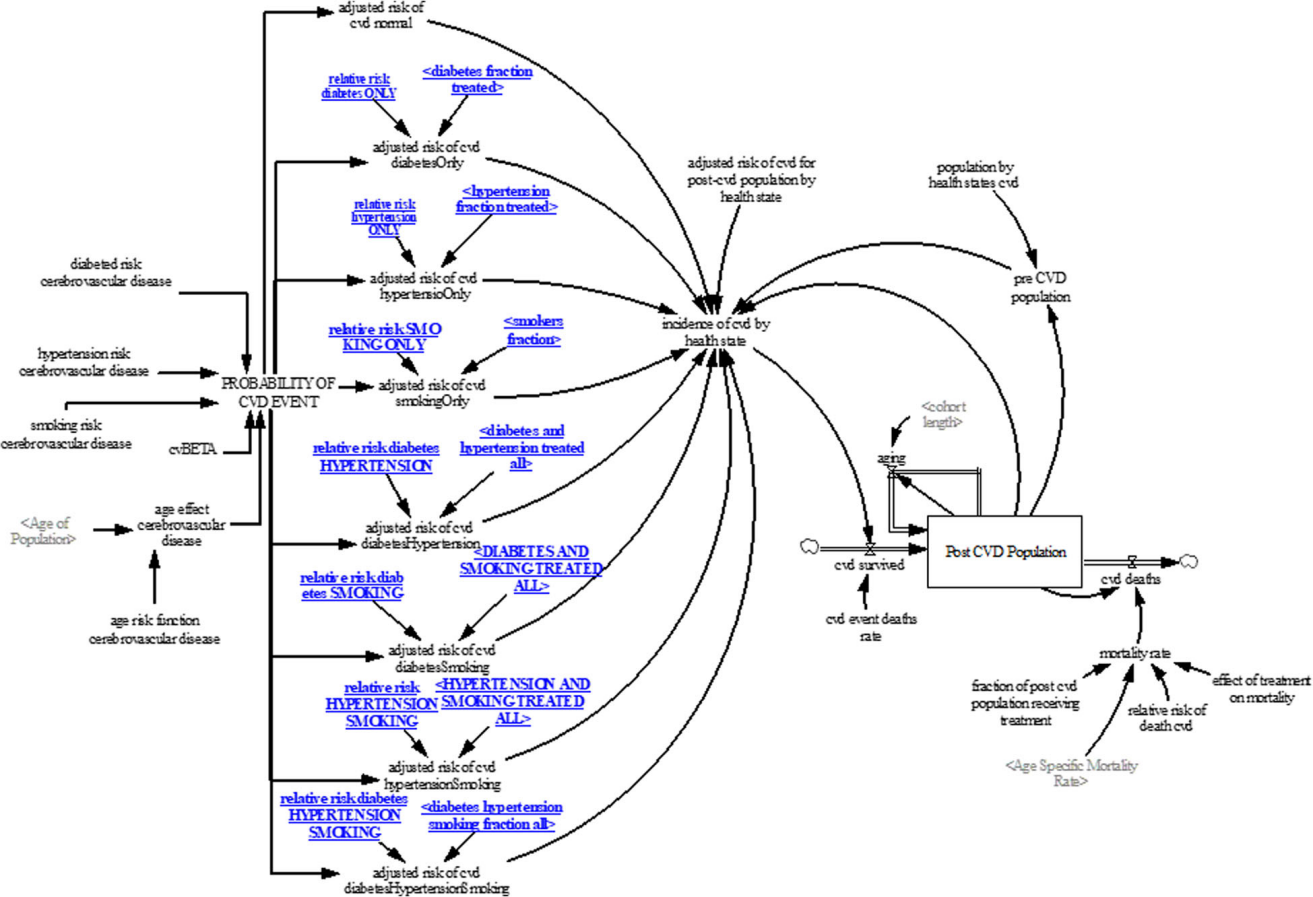
Cerebrovascular disease sub-model

The CVD sub-model (see Fig. 3) projects CVD events, CVD deaths, and post-CVD population of Singapore 18 years and older. The Framingham Risk Engine for stroke [57, 58] was used as a foundation to estimate the probability of CVD events given the risk factors. To estimate the probability of CVD events, the population was divided into eight risk groups to allow for estimating different CVD event probabilities for each risk group. Each risk factor was evenly distributed across the number of risk groups due to the lack of data on comorbidity of risk factors. The risk groups are (a) normal—individuals with no known risk factors; (b) diabetes only; (c) hypertension only; (d) smoking only; (e) diabetes and hypertension; (f) diabetes and smoking; (g) hypertension and smoking; (h) diabetes, hypertension and smoking. The equations for the probability of CVD events given the risk group are:

1 $$ P\left({CVD}_{normal}\right)=\raisebox{1ex}{$1$}\!\left/ \!\raisebox{-1ex}{$\left(1+ EXP\left(-\left({\beta}_0+{\beta}_1{age}_i\right)\right)\right)$}\right. $$

2 $$ P\left({CVD}_{diabetesOnly}\right)=\raisebox{1ex}{$1$}\!\left/ \!\raisebox{-1ex}{$\left(1+ EXP\left(-\left({\beta}_0+{\beta}_1{age}_i+{\beta}_2 diabetes\right)\right)\right)$}\right. $$

3 $$ P\left({CVD}_{hypertensio\mathrm{n} Only}\right)=\raisebox{1ex}{$1$}\!\left/ \!\raisebox{-1ex}{$\left(1+ EXP\left(-\left({\beta}_0+{\beta}_1{age}_i+{\beta}_3 hypertenion\right)\right)\right)$}\right. $$

4 $$ P\left({CVD}_{smokinOnly}\right)=\raisebox{1ex}{$1$}\!\left/ \!\raisebox{-1ex}{$\left(1+ EXP\left(-\left({\beta}_0+{\beta}_1{age}_i+{\beta}_4 smoking\right)\right)\right)$}\right. $$

5 $$ P\left({CVD}_{diabetes\& hypertension}\right)=\raisebox{1ex}{$1$}\!\left/ \!\raisebox{-1ex}{$\left(1+ EXP\left(-\left({\beta}_0+{\beta}_1{\mathrm{a} ge}_i+{\beta}_2 diabetes+{\beta}_3 hypertenion\right)\right)\right)$}\right. $$

6 $$ P\left({CVD}_{diabetes\& smoking}\right)=\raisebox{1ex}{$1$}\!\left/ \!\raisebox{-1ex}{$\left(1+ EXP\left(-\left({\beta}_0+{\beta}_1{age}_i+{\beta}_2 diabetes+{\beta}_4 smoking\right)\right)\right)$}\right. $$

7 $$ P\left({CVD}_{hypertension\& smoking}\right)=\raisebox{1ex}{$1$}\!\left/ \!\raisebox{-1ex}{$\left(1+ EXP\left(-\left({\beta}_0+{\beta}_1{age}_i+{\beta}_3 hypertenion+{\beta}_4 smoking\right)\right)\right)$}\right. $$

8 $$ P\left({CVD}_{diabetes\& hypertension\& smoking}\right)=\raisebox{1ex}{$1$}\!\left/ \!\raisebox{-1ex}{$\left(1+ EXP\left(-\left({\beta}_0+{\beta}_1{age}_i+{\beta}_2 diabetes+{\beta}_3 hypertenion+{\beta}_4 smoking\right)\right)\right)$}\right. $$

Where *β* is the coefficient; *β*_0_ is the intercept ;*β*_1_ is the coefficient for age; *β*_2_ is the coefficient for diabetes; *β*_3_ is the coefficient for hypertension; and *β*_4_ is the coefficient for smoking. The coefficients (*β*_1_, *β*_2_, *β*_3_, *β*_4_) were derived from the Framingham Risk Engine as cited [57, 58], while the coefficient *β*_0_ was derived through calibration. Due to lack of data at the population level, a table (see Table 1) of the likely distribution of CVD patients by risk groups was generated by a senior physician with significant experience in CVD care. This table allows us to distribute the CVD events data into CVD events by risk groups to calibrate the model to obtain *β*_0_ for all risk groups.

**Table 1:** Distribution of CVD events by risk group by an expert

	No risk	Diabetes	Hypertension	Smoking	Diabetes Hypertension
No risk	1%				
Diabetes		20%	29%	5%	
Hypertension			10%	15%	
Smoking				5%	15%

Evidence from many studies [7–11] suggests that CVD events can be significantly reduced if hypertension and diabetes patients are treated to target. To account for the effect of hypertension and diabetes treatment on CVD events, the probability of CVD events is adjusted by the proportion of the population in each risk group receiving treatment (in the case of smoking we consider treatment as smoking cessation) and the effect of treatment on CVD event probability (i.e., the relative probability of CVD event due to treatment). An example of an adjusted probability for individuals with diabetes only is:

10 $$ AP\left({CVD}_{diabetes}\right)=\left(P\left({CVD}_{diabetes}\right)\ast \left(1-\left(\raisebox{1ex}{${fd}_{tot}$}\!\left/ \!\raisebox{-1ex}{${fd}_0$}\right.\right)\right)\right)+\left(P\left({CVD}_{diabetes}\right)\ast \left(\raisebox{1ex}{${fd}_{tot}$}\!\left/ \!\raisebox{-1ex}{${fd}_0$}\right.\right)\ast \left(1-{RR}_d\right)\right)\kern0.5em $$

Where *AP*(*CVD*_*diabetes*_) is the adjusted probability of CVD events for patients with diabetes; *P*(*CVD*_*diabetes*_) is the CVD event probability among the diabetic risk group; *fd*_*tot*_ is the fraction of the patients in the diabetic only risk group receiving treatment; *fd*_0_ is the fraction of the population with diabetes only; *RR*_*d*_ is the relative risk of treatment for diabetic on CVD event. To estimate CVD events, the adjusted probability of CVD event is multiplied by the number of people in each risk factor group to determine the number of CVD events per year. Adjusted CVD event probabilities are then applied to the risk groups to obtain CVD events for each risk group.

To account for the impact of CVD events on the number of people living with CVD associated disabilities, a stock and flow structure was developed. The CVD disability population increases as people survive CVD events and decreases through CVD-related deaths. The CVD survival population is estimated by the number of CVD events and CVD event deaths rate derived from available data [59].

#### Data

The demographic dataset used as input for the risk factor sub-models was obtained from the Singapore Statistics department [47]. Time series data regarding the prevalence of diabetes, hypertension, smoking and CVD events was obtained from the National Registry of Diseases [60]. Lastly, the *Framingham Risk Engine* data was obtained from literature [57]. The data sources and input parameters used are listed in Table 2.

**Table 2:** Model Parameters

Parameters	Values	Unit	Sources
*Diabetes sub model*			
Baseline incidence rate	0.0328368	Dimensionless/year	Calibration
Baseline progression rate [managed]	0.0134	Dimensionless/year	[49]
Baseline progression rate [unmanaged]	0.0556	Dimensionless/year	
Baseline pre-diabetes regression rate	0.193	Dimensionless/year	[50]
Uptake rate of management	0.05	Dimensionless/year	Calibration
*Hypertension sub-model*			
Baseline incidence rate	0.0237906	Dimensionless/year	Calibration
Baseline progression rate [unmanaged]			[51]
Female	0.06975	Dimensionless/year	
Male	0.080125	Dimensionless/year	
Effect of management on progression	0.33	Dimensionless/year	[52]
Baseline prehypertension regression rate	0.25	Dimensionless/year	[53]
Uptake rate of management	0.035	Dimensionless/year	Calibration
*Smoking sub-model*			
Baseline smoking initiation rate	0.0082388	Dimensionless/year	Calibration
Baseline smoking relapse rate	0.1	Dimensionless/year	[56]
Baseline smoking cessation rate	0.162	Dimensionless/year	[55]
*Demographics*			
Birth rate	Time series	Dimensionless/year	[47]
Net migration	Time series	Dimensionless/year	Calibration
Age specific mortality rate	Time series	Dimensionless/year	[47]
*CVD Model*			
Smoking risk			[58]
Female	0.5419	Dimensionless	
Male	0.5224	Dimensionless	
Age risk			
Female	0.0699	Dimensionless	
Male	0.0488	Dimensionless	
Diabetes risk			
Female	0.5604	Dimensionless	
Male	0.3492	Dimensionless	
Hypertension risk			
Female	0.0161	Dimensionless	
Male	0.0152	Dimensionless	
CVD beta			Calibration
Normal	−11.968	Dimensionless	
Diabetes only	−5.53323	Dimensionless	
Hypertension only	−6.91289	Dimensionless	
Smoking only	−5.33926	Dimensionless	
Diabetes and hypertension	−6.74229	Dimensionless	
Diabetes and smoking	−8.09581	Dimensionless	
Hypertension smoking	−7.10026	Dimensionless	
Diabetes and hypertension and smoking	−8.18119	Dimensionless	
Relative risk of CVD event Diabetes Management	0.8	Dimensionless	[61]
Relative risk of CVD event Hypertension Management	0.5	Dimensionless	[62]
Relative risk of CVD event Smoking Cessation	0.5	Dimensionless	Calibrated
CVD event death rate	0.2	Dimensionless	Calibrated
Relative risk of death CVD	1.6	Dimensionless	[63]
Effect of treatment on mortality	0.13	Dimensionless	[67]
Fraction of post-CVD population receiving treatment of risk factors	1	Dimensionless	Expert opinion

#### Model validation and sensitivity analysis

The structure and behavioural validation test [38] was used to validate the model. On the structure test, the model was presented to clinicians with expert knowledge on CVD to verify the model structure and its assumptions regarding causal relationships. Therefore, the model is grounded on current knowledge and available evidence on the risk factors of CVD and interventions to prevent CVD events. The behaviour test shows simulated behaviour of the prevalence of the key variables of diabetes, hypertension, smoking and CVD, in comparison with available data (see Additional file 1: Figure S1 for behaviour validation graphs). The results suggest that the simulated model behaviour compares favourably with data, indicating that the model performs credibly for visual fit test.

For the sensitivity analysis, a two-way sensitivity analysis was performed on the base-case and the policy experiments to evaluate the likely impact of how a change in the most important parameters affects CVD outcomes. The most important parameter included in the sensitivity analysis was incidence rate for diabetes, hypertension, and smoking initiation. These rates were varied ±50%, and the model was run 500 times. The estimated average and the minimum and maximum values at 95% confidence level for each run, were used to show the credible interval.

#### Policy experimentation

##### Base-case

The base-case experiment assumes no change to key parameters that may be affected by policy change, such as incidence and management uptake of hypertension and diabetes, and smoking initiation and cessation. Although this hypothetical experiment assuming these factors to be constant is unlikely in the current context due to the changeable nature of public health policies, it is included to serve as a reference point for evaluating the alternative policy experiments.

##### Diabetes Management Scenario

In this scenario, the uptake rate of diabetes management is assumed to increase from 5 to 15% in 2020 and remain unchanged over the simulation time

##### Hypertension Management Scenario

In this scenario, the uptake rate of hypertension management among hypertensive patients is assumed to increase from 3.5 to 13.5% in 2020 and remains constant to 2040.

##### Smoking Cessation Scenario

In this scenario, the smoking cessation rate among the smoking population is assumed to increase from 16.2 to 25% in 2020 and remains constant over the simulation time.

##### Diabetes/Hypertension/Smoking Scenario

This scenario implements the diabetes, hypertension and smoking scenarios concurrently to assess its impacts on CVD outcomes.

## Results

### Risk factors

Due to an aging population and lifestyle changes, the number of Singaporeans with diabetes is projected to increase 142% from about **369,133** in 2010 to **893,412** [95% confidence interval: 880,079-906,745] by 2040 in the base-case scenario. The number of people with hypertension is projected to increase from **659,958** in 2010 to **1,067,049** (1,053,627-1,080,470) by 2040, representing 61.6% increase. The number of Singaporean smokers 18 years and older is projected to increase by 17% from **414,789** in 2010 to **485,524** (480,426-490,622) by 2040. In the hypothetical scenarios experimented, the proportion of diabetes patients under management is projected to increase from 44 to 81% by 2040; whereas hypertension patients under management is projected to rise from 43 to 84% by 2040 and the proportion of the population using tobacco (smoking) is projected to decrease from 13 to 9.2% by 2040.

#### CVD outcomes

Table 3 shows the results of the CVD outcomes. Under the base-case scenario, the number of CVD event (incidence of CVD) is projected to increase by 160% from 33,292 in 2010 to 86,592 (85,588-87,594) by 2040. Consequently, the projected number of death associated with the CVD events is projected to increase from 5329 in 2010 to 14,714 (14,631-14,797) in 2040, representing 176% increase. The post-CVD population in Singapore is projected to increase by 81.7% from 112,606 in 2010 to 204,689 (202,857-206,522) by 2040. Also, the age-adjusted incidence rate of CVD is projected to increase from 0.88% in 2010 to 1.84% by 2040. In the diabetes management scenario—where the proportion of individuals with diabetes managed is assumed to increase from 44 to 81% after 2020 and remains unchanged over the simulation time—the CVD events is projected to be 75,647 (74,819-76,475), representing a 12.6% reduction by 2040 compared to the base-case scenario, while CVD deaths is projected to decrease 5.7% by 2040, relative to the base-case scenario. Correspondingly, the post-CVD population is projected to be 186,460 (184,923-187,997), representing an 8.9% reduction by 2040, as compared to the base-case scenario. Likewise, the age-adjusted incidence rate of CVD is project to decrease by 12.5% by 2040 compared to the base-case scenario. In the hypertension management scenario—where the proportion of individuals under management is assumed to increase from 43 to 84% after 2020 and remains unchanged—the CVD events is projected to be 79,812 (78,912-80,712), representing a decrease of 7.8% by 2040 compared to the base-case scenario; whereas the CVD deaths and post-CVD population are projected to decrease 3.9 and 4.8% respectively, relative to the base-case scenario; whereas the age-adjusted incidence rate of CVD is projected to decrease by 7.6% relative to the base-case scenario in 2040. In the scenario where smoking cessation is implemented, it is assumed that smoking prevalence will decrease from 13% in 2020 to 9.6% by 2040, resulting in CVD events and deaths decreasing by 4.2 and 2.6% respectively, as compared to the base-case scenario. Additionally, the post-CVD population is projected to decrease by 3.8% compared to the base-case scenario; whereas the age-adjusted incidence rate of CVD is projected to decrease by 3.9% in 2040, compared to the base-case scenario. Lastly, under the combined scenario where diabetes, hypertension and smoking cessation management policies are implemented concurrently, CVD events, CVD deaths and post-CVD population are projected to decrease by 24, 12.2 and 17.4% respectively, compared to the base-case scenario. Lastly, the age-adjusted incidence rate of CVD is projected to decrease by 24.1% in 2040, relative to the base-case scenario.

**Table 3:** CVD Outcomes

Outcomes	2010	2020	2030	2040	% change 2010–2040
Base-case					
CVD Events	33,292 (33,292-33,292)	52,171 (51,982-52,360)	70,102 (69,542-70,662)	86,592 (85,588-87,594)	160% (157–163%)
CVD Deaths	5329 (5329-5329)	7046 (7043-7049)	10,551 (10,528-10,574)	14,714 (14,631-14,797)	176% (174–177.6%)
Post-CVD Population	112,606 (112,606-112,606)	138,507 (138,366-138,647)	173,896 (173,137-174,654)	204,689 (202,857-206,522)	81.7% (80–83.4%)
Age-Adjusted Incidence Rate	0.88% (0.88–0.88%)	1.293% (1.289–1.298%)	1.60% (1.59–1.61%)	1.84% (1.82–1.86%)	109% (106–111%)
Diabetes Management Scenario					
CVD Events	33,292 (33,292-33,292)	52,171 (51,982-52,360)	63,857 (63,391-64,322)	75,647 (74,819-76,475)	127% (124–129%)
CVD Deaths	5329 (5329-5329)	7046 (7043-7049)	10,331 (10,311-10,352)	13,875 (13,804-13,946)	160% (159–161%)
Post-CVD Population	112,606 (112,606-112,606)	138,507 (138,366-138,647)	168,088 (167,410-168,766)	186,460 (184,923-187,997)	65.5% (64–66.9%)
Age-Adjusted Incidence Rate	0.88% (0.88–0.88%)	1.293% (1.289–1.298%)	1.46% (1.45–1.47%)	1.61% (1.59–1.62%)	82.9% (80.6–84%)
Hypertension Management Scenario					
CVD Events	33,292 (33,292-33,292)	52,171 (51,982-52,360)	66,500 (65,979-67,021)	79,812 (78,912-80,712)	139.7% (137–142%)
CVD Deaths	5329 (5329-5329)	7046 (7043-7049)	10,383 (10,361-10,405)	14,130 (14,053-14,206)	165% (163.7–166%)
Post-CVD Population	112,606 (112,606-112,606)	138,507 (138,366-138,647)	170,720 (169,991-171,449)	194,710 (193,019-196,401)	71.9% (71.4–74%)
Age-Adjusted Incidence Rate	0.88% (0.88–0.88%)	1.293% (1.289–1.298%)	1.52% (1.51–1.53%)	1.70% (1.68–1.72%)	93% (90–95%)
Smoking Cessation Scenario					
CVD Events	33,292 (33,292-33,292)	52,171 (51,982-52,360)	67,036 (66,505-67,567)	82,973 (82,013-83,933)	149% (146–152%)
CVD Deaths	5329 (5329-5329)	7046 (7043-7049)	10,413 (10,391-10,434)	14,322 (14,243-14,400)	168% (167–170%)
Post-CVD Population	112,606 (112,606-112,606)	138,507 (138,366-138,647)	170,178 (169,451-170,905)	196,800 (195,048-198,551)	74.7% (73–76%)
Age-Adjusted Incidence Rate	0.88% (0.88–0.88%)	1.293% (1.289–1.298%)	1.536% (1.524–1.548%)	1.768% (1.748–1.788%)	100.9% (98–103%)
Diabetes/Hypertension/Smoking Scenario					
CVD Events	33,292 (33,292-33,292)	52,171 (51,982-52,360)	57,347 (56,947-57,747)	65,584 (64,897-66,271)	96.9% (94.9–99%)
CVD Deaths	5329 (5329-5329)	7046 (7043-7049)	10,029 (10,010-10,048)	12,918 (12,856-12,979)	142% (141–143.5%)
Post-CVD Population	112,606 (112,606-112,606)	138,507 (138,366-138,647)	161,321 (160,702-161,939)	169,112 (167,789-170,434)	50% (49–51.3%)
Age-Adjusted Incidence Rate	0.88% (0.88–0.88%)	1.293% (1.289–1.298%)	1.313% (1.304–1.322%)	1.396% (1.381–1.410%)	58.6% (56%-60)

## Discussion

In response to population increase and aging, the number of Singaporeans with diabetes and hypertension is projected to surge. Concurrently, the smoking population is projected to rise. As a result, CVD events, CVD deaths, and post-CVD Singaporean population 18 years and older is projected to increase. The results from the quantitative simulation suggest that based on the current demographic composition of Singapore and available evidence from randomized control trials on the impact of diabetes, hypertension and smoking cessation interventions on CVD [7–11, 61], aggressive management of diabetes will have the most impact on CVD outcomes, in comparison with aggressive management of hypertension only or smoking cessation management intervention only. Moreover, smoking intervention alone will have the least impact on CVD outcomes compared to diabetes and hypertension management interventions.

The insight that despite the fact that at the individual level, hypertension management is associated with highest risk reduction for CVD (50%) in comparison to diabetes and smoking (20%) while being the most prevalent risk factor, at the population level, diabetes management interventions are projected to have higher impact on reducing CVD events when compared to hypertension management or smoking cessation interventions. This is because the risk of CVD events associated with diabetes is significantly higher (0.5604 for females and 0.3492 for males) than those associated with hypertension (0.0161 for female and 0.0152 for males); hence, the adjusted risk for CVD events at the population is expected to reduce significantly with diabetes management intervention rather than hypertension management intervention. For smoking, although the risk of CVD events associated with smoking (0.5419 for females and 0.5224 for males) is comparable to that of diabetes, diabetes management interventions are expected to be significantly more effective for improving CVD outcomes relative to that of smoking cessation management intervention due to the rate of smoking being projected to increase moderately (17%) compared to that of diabetes (139%).

This insight suggests that CVD outcomes are likely to improve if policymakers place more emphasis on identifying and managing individuals with hypertension and diabetes. Policymakers must thus be increasingly proactive in implementing chronic disease policies that prioritize care continuity, with emphasis on screening, and effective management of hypertension and diabetes, in order to reduce the incidence, death and disability associated with CVD. In addition, policies focusing on educating the public to adopt a healthy lifestyle to reduce the risk of chronic diseases should be promoted, such as the emphasis of the importance of regular exercise, maintaining a healthy diet and abstaining from tobacco products. Singapore has declared war on diabetes [64] due to poor diabetic outcomes compared to countries in the OECD. Though a noble initiative, the finding from the simulation study suggests that the war on diabetes should be expanded to include a war on hypertension in order to significantly increase the accrued health benefits to Singapore, as infrastructural setup for improving diabetes care could simultaneously be used to care for people with hypertension. Furthermore, most patients with diabetes tend to have hypertension [65, 66], further emphasizing the need to care for both conditions.

The strength of this paper lies in the use of aggregate data at the population level to estimate the impact of diabetes and hypertension management and smoking cessation on CVD outcomes. Estimation of the impact of diabetes and hypertension management and smoking cessation will then allow policymakers and care providers to estimate the number of people who are likely to suffer from CVDs and allocate the resources required to provide adequate services to meet the health needs of the society. In addition, policy makers can now project the budget impact of interventions by estimating the number of individuals with diabetes and hypertension that need to be managed over time and the cost for management. However, the simulation model presented herein has some limitations. First, only individuals 18 years and older were included in the model, leading to a likely underestimation of the number of people with the risk factors of diabetes, hypertension, and smoking. Second, due to lack of data, we assumed an even distribution of individuals with risk factors into the health state of individuals with comorbidity. Lastly, the risk engine used in the model is from non-Asian population, which could lead to an over or under estimation of the probability of CVD events. Although a Singapore specific risk engine is currently unavailable, future models should make the effort to include such a risk engine as the populations are likely to differ.

## Conclusion

As a result of an aging population and the increasing prevalence of chronic conditions, the number of CVD events in Singapore is projected to rise significantly in the near future. The findings from this research synthesize data from a variety of sources and suggest that CVD events and associated deaths and disabilities could be reduced significantly if diabetes and hypertension patients are aggressively managed. Although health policy in Singapore is increasing emphasis on the prevention and management of chronic conditions, a recent noble policy that declared a war on diabetes should be expanded to include a war on hypertension in order to fully benefit from the combined impact of diabetes and hypertension management.

## Additional file

Additional file 1: **Figure S1.** Behavior validation graphs. (TIF 124 kb)

## Data Availability

The datasets used in the manuscript are publically available and the references and links to the data are provided below: I. SingStat. Population Trends 2017 [Internet]. 2018. Available from: https://www.singstat.gov.sg/publications/population-trends II. SingStat. Monthly Household Income from Work Including Employer CPF Contributions. [Internet]. Available from: http://www.tablebuilder.singstat.gov.sg III. Nichols GA, Hillier TA, Brown JB. Progression from newly acquired impaired fasting glucose to type 2 diabetes. Diabetes Care. 2007;30(2):228–33. IV. Fonseca VA. Identification and treatment of prediabetes to prevent progression to type 2 diabetes. Clin Cornerstone. 2007;8(2):10–20. V. Kim SJ, Lee J, Nam CM, Jee SH, Park IS, Lee KJ, et al. Progression Rate From New-Onset Pre-Hypertension to Hypertension in Korean Adults. Circ J [Internet]. 2011;75(1):135–40. Available from: http://joi.jlc.jst.go.jp/JST.JSTAGE/circj/CJ-09-0948?from=CrossRef VI. Chobanian A V. Prehypertension revisited. Hypertension. 2006;48(5):812–4. VII. Neaton JD. Treatment of Mild Hypertension Study. JAMA [Internet]. 1993 Aug 11;270(6):713. Available from: http://jama.jamanetwork.com/article.aspx?doi=10.1001/jama.1993.03510060059034 VIII. Ministry of Health Singapore, National Health Survey 2004 [Internet]. 2005. Available from: https://www.moh.gov.sg/resources-statistics/reports/national-health-survey-2004 IX. García-Rodríguez O, Secades-Villa R, Flórez-Salamanca L, Okuda M, Liu SM, Blanco C. Probability and predictors of relapse to smoking: Results of the National Epidemiologic Survey on Alcohol and Related Conditions (NESARC). Drug Alcohol Depend [Internet]. 2013;132(3):479–85. Available from: 10.1016/j.drugalcdep.2013.03.008 X. D’Agostino RB, Vasan RS, Pencina MJ, Wolf PA, Cobain M, Massaro JM, et al. General cardiovascular risk profile for use in primary care: The Framingham heart study. Circulation. 2008;117(6):743–53. XI. Lackland DT, Carey RM, Conforto AB, Rosendorff C, Whelton PK, Gorelick PB. Implications of recent clinical trials and hypertension guidelines on stroke and future cerebrovascular research. Stroke. 2018;49(3):772–9. XII. Patel A. Effects of a fixed combination of perindopril and indapamide on macrovascular and microvascular outcomes in patients with type 2 diabetes mellitus (the ADVANCE trial): a randomised controlled trial. Lancet [Internet]. 2007 Sep;370(9590):829–40. Available from: http://linkinghub.elsevier.com/retrieve/pii/S0140673607613038 XIII. Wannamethee SG, Shaper AG, Lennon L. Cardiovascular disease incidence and mortality in older men with diabetes and in men with coronary heart disease. Heart. 2004;90(12):1398–403. XIV. Prevention of Stroke by Antihypertensive Drug Treatment in Older Persons With Isolated Systolic Hypertension: Final Results of the Systolic Hypertension in the Elderly Program (SHEP). *JAMA.*1991;265(24):3255–3264. doi:10.1001/jama.1991.03460240051027 XV. https://www.nrdo.gov.sg/

## Abbreviations

CVD: Cardiovascular diseases
EXP: Exponent
HbA1C: Haemoglobin A1C
mmHg: Millimetre of mercury
PRISM: Prevention Impacts Simulation Model
UK: United Kingdom
US: United States
