# Supplementary figures and images for: An evaluation of the impact of aggressive hypertension, diabetes and smoking cessation management on CVD outcomes at the population level: a dynamic simulation analysis
Source: BMC Public Health. 2019 Aug 14;19:1105. doi: 10.1186/s12889-019-7429-2 (PMC6694535; doi:10.1186/s12889-019-7429-2)

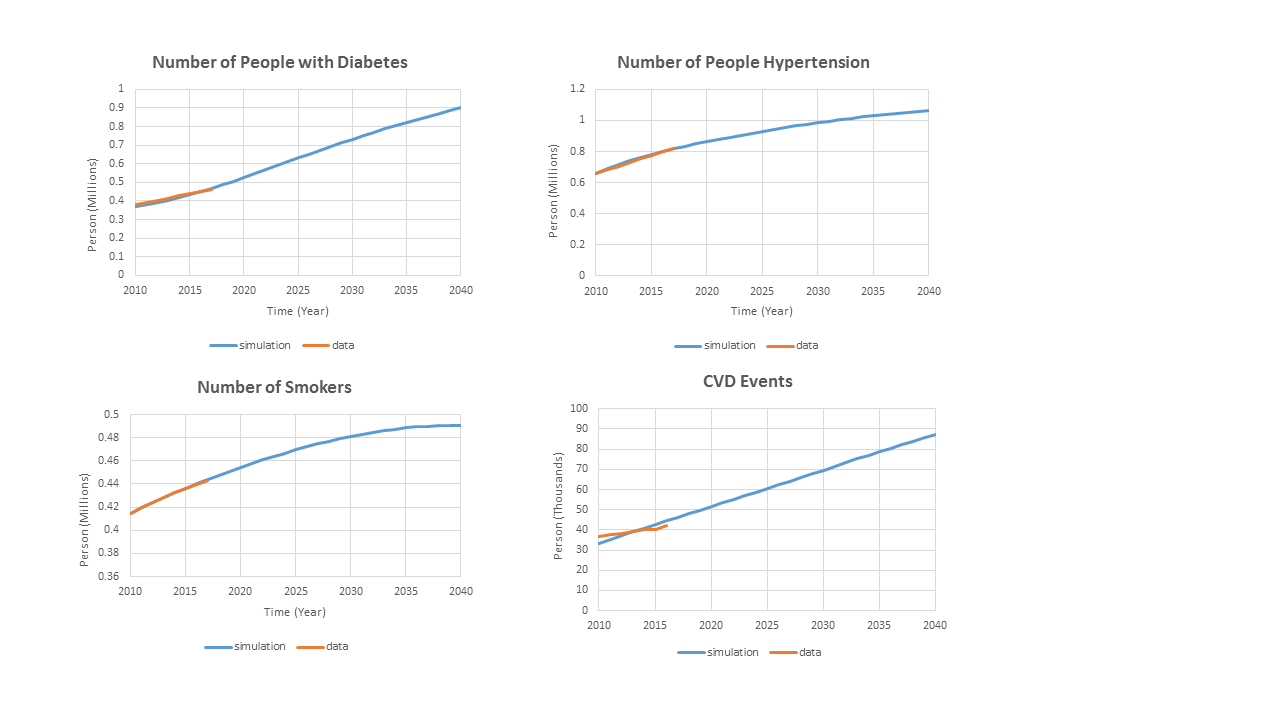

Supplement: Supplementary file 1 — Figure S1. Behavior validation graphs. (TIF 124 kb) [file 12889_2019_7429_MOESM1_ESM.tif]
